# Effect on bone mineralization of continued growth hormone therapy at the transition between childhood and adulthood

**DOI:** 10.1530/EC-25-0346

**Published:** 2026-01-05

**Authors:** C Bailly, E Le Roux, M Polak, P Touraine

**Affiliations:** ^1^ Endocrinology and Reproductive Medicine Department, Pitié-Salpêtrière University Hospital, AP-HP, Paris, France; ^2^ Center for Rare Disorders CRESCENDO, Paris, France; ^3^ Unit of Clinical Epidemiology, Hôpital Universitaire Robert-Debré, Assistance Publique des Hôpitaux de Paris (AP-HP), Paris, France; ^4^ Pediatric Endocrinology, Diabetology, and Gynecology Department, Necker-Enfants Malades University Hospital, AP-HP, Paris, France; ^5^ Université Paris Cité, Paris, France; ^6^ Sorbonne University, Paris, France

**Keywords:** growth hormone deficiency, growth hormone therapy, bone density, transition between childhood and adulthood

## Abstract

**Abstract:**

Childhood-onset growth-hormone deficiency (GHD) requires growth-hormone therapy (GHT) to optimize final height. GHT may also have benefits after final height is achieved, notably on bone. The objective of this retrospective single-center observational cohort study of patients who had persistent GHD after achieving their final height was to assess bone parameters at the transition to adult care and then more than 6 months later (6.08 (3.33–10.25) years). Bone mineral density (BMD) and Z-score at the lumbar spine and hip were determined. Of 162 patients transitioned to adult care in 1994–2021, 105 received GHT for longer than 6 months (GHT group) and 57 received either no GHT or GHT for less than 6 months (no-GHT group); however, BMD and Z-score data from both evaluations were available for only a minority of patients. Lumbar-spine BMD was significantly higher at the second evaluation in the GHT group than in the no-GHT group (*P* = 0.034). The lumbar-spine Z-scores at the first and second evaluations were −1.61 and −1.32 in the no-GHT group vs −1.09 and −0.61 in the GHT group (*P* = 0.047 for the difference in median change over time between groups). Changes in BMD at the lumbar spine and in BMD and Z-score at the femoral neck were not significantly different between the two groups. These results suggest beneficial effects of continued GHT in patients with persistent GHD after final height attainment, adding evidence to continue GH therapy during and after the transition period.

**Plain language summary:**

Children with insufficient growth-hormone levels are given growth-hormone injections to allow them to gain height. Once the final height is achieved, the treatment is often stopped. However, further treatment during the transition to adulthood may have benefits, notably on bone.

## Introduction

Growth hormone deficiency (GHD) is a rare disease with a prevalence of 1/1,107 to 1/8,646 (1). Onset may occur during childhood or adulthood. The diagnosis is challenging for clinicians and for patients and their families. In childhood-onset GHD (CoGHD), replacement growth hormone therapy (GHT) is essential to achieve the optimal final height. CoGHD may persist into adulthood.

GHT is a costly intervention whose continuation into adulthood remains controversial due to a scarcity of long-term data (2). Until recently, achieving a satisfactory final height was viewed as the main goal of GHT during childhood, which was therefore often stopped upon the completion of puberty. However, patients with CoGHD who have achieved their final height have lower bone mass, lower muscle mass, and higher fat mass compared to healthy controls and may therefore be at higher risk for cardiovascular disease and osteoporosis later in life (3, 4). Bone mineral accrual continues until at least 25 years of age, and a sufficient supply of GH is important to obtain the optimal peak bone mass (5). GHT in adulthood has beneficial effects on metabolic parameters, bone mass, and quality of life (6, 7). GHT given in adult-onset GHD was followed by bone mineral density (BMD) gains in several studies (8, 9, 10, 11, 12). Data on GHT after final height achievement in patients with CoGHD are scarcer. However, a randomized trial in 160 patients with CoGHD aged 18–25 years found that continuing GHT for 2 years significantly increased lumbar-spine and hip BMD (13). A narrative review also supported continuing GHT in patients who had persistent GHD after attaining their final height (5). Guidelines issued in 2019 recommend GHT at the transition to adulthood if tests show persistent GHD (14). Other guidelines indicate that GHT should be continued until peak bone mass is achieved but do not specify the criteria for assessing this outcome, especially the age (5). Individual dosage optimization has also been suggested (3). Given the imprecision and short follow-up of these data and the high cost of GHT, deciding whether GHT given to treat CoGHD should be continued during the transition from pediatric to adult healthcare is challenging (15). The risk/benefit ratio must be assessed thoroughly, based not only on objective clinical variables but also on the patient’s quality of life. Importantly, the transition to adulthood is a difficult period marked by massive social and psychological changes, even in the absence of disease. In patients with chronic childhood-onset diseases, interruptions in treatments and missed follow-up appointments are common at the transition (16). Adherence may be particularly difficult to obtain with GHT, given the need for daily injections. Moreover, final height is usually the main concern for patients with CoGHD, who may therefore lack the motivation needed to continue GHT once statural growth is complete.

Convincing patients that continued GHT is desirable to gain less palpable advantages compared to sufficient height, such as good metabolic control and optimal peak bone mass, can be extremely difficult. Thus, a survey found suboptimal adherence in 64–77% of patients depending on the range of age (one group of adults, one group of adolescents from 13 to 17 years old, and one group of parents of children from 4 to 12 years old) (17). The availability of sound data on the benefits of continued GHT would help to convince patients of the importance of good adherence.

Our group previously reported BMD findings in 112 patients with CoGHD who transitioned to adult care in 1994–2012 (7). In this retrospective observational study, most patients (83%) stopped GHT before the transfer, at a median of 16.3 years. Median age at transfer was 19.4 years. After the transfer, 101 patients who had stopped GHT had persistent GHD. A comparison between patients with persistent GHD who received GHT and those who did not, with evaluations conducted approximately 2.5 years apart, showed that BMD at the spine and hip increased significantly only in the GHT group but that the differences with the no-GHT group were not statistically significant.

The objective of the present single-center retrospective observational study was to further assess BMD changes at the transition in patients with CoGHD, according to whether GHT was stopped or continued into adulthood, with a larger cohort and longer follow-up.

## Materials and methods

### Study design and population

We conducted a retrospective observational single-center cohort study of consecutive patients with CoGHD transferred from pediatric care to our adult-care endocrinology department at the Pitié-Salpêtrière University Hospital (Paris, France) between January 1, 1994, and September 1, 2021. The cohort combines the patients in the previous study (7) and the patients transferred to the same adult-care department between 2013 and 2021.

In accordance with French law on retrospective analyses of de-identified health data, neither ethics committee approval nor informed patient consent was required for this study.

The inclusion criteria were CoGHD with GHT during childhood, GHD documented during the first evaluation (EVAL1) done in the adult-care department, and available data from a second evaluation (EVAL2) done at least 6 months after EVAL1. Both evaluations had to have been done during a day hospitalization in the adult-care department. All patients meeting these inclusion criteria were enrolled in the study.

### Evaluations and care in the adult department

After the transfer and at least 1 month after GHT discontinuation, hormonal provocative tests (insulin tolerance test) were performed to look for persistent GHD, which was an inclusion criterion. GHD was defined as a peak GH response below 15 mIU/L or 5 μg/L (18).

We separated the patients into two groups depending on whether they did or did not receive GHT: CoGHD patients continuing GH treatment for more than 6 months between EVAL1 and EVAL2 (GHT), and CoGHD patients receiving 6 months or less of GH treatment between EVAL1 and EVAL2 (no GHT).

### Data collection

The study data were retrieved from the medical records of each patient at the time of the second evaluation. In addition to age and sex, for each patient we recorded the following data obtained during each evaluation: body weight (kg), height (meters), body mass index (BMI, kg/m^2^), metabolic tests (total cholesterol, HDL cholesterol, LDL cholesterol, triglycerides, fasting blood glucose, and HbA1c), insulin-like growth factor-1 (IGF-1), and BMD and Z-score at the femoral neck and lumbar spine. IGF-1 was measured using an immunoradiometric assay (CIS Bio, France) until 2010 and a chemiluminescence assay (Liasion XL Diasorin, Anthony, France) thereafter. The IGF-1 values were adjusted for age and sex. The BMD data were obtained using dual-energy X-ray absorptiometry (Hologic Discovery™, Hologic, USA) and recorded in g/cm^2^ and, given the age of the population, as Z-scores.

### Statistical analyses

Quantitative variables were described as median (first quartile–third quartile) and categorical variables as numbers (frequencies). We used the Shapiro test to assess data normality and the Levene test for homogeneity of variances. Normally distributed variables with homogeneous variance were compared by applying Student’s *t*-test; other were compared using the Mann–Whitney test. For BMI-adjusted analyses, we used the GLM test.

The statistical analyses were done using *jamovi* (version 2.6; https://www.jamovi.org). *P* values smaller than 0.05 were taken to indicate significant differences. We did not take into account the missing data.

## Results

### Population

**Figure 1:**
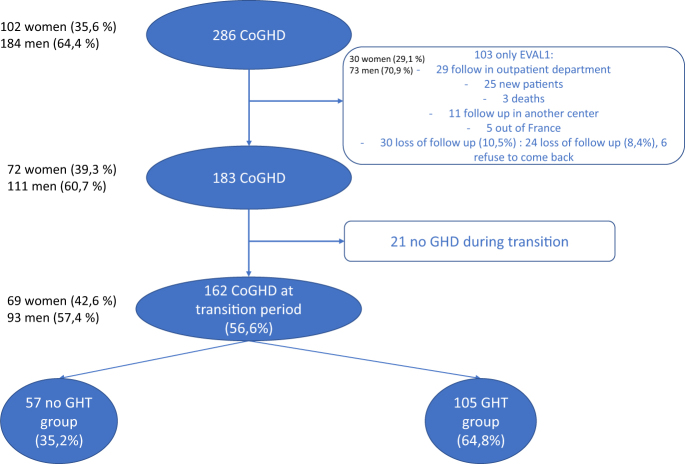
Patient flowchart. CoGHD: childhood-onset growth-hormone deficiency; EVAL1: first evaluation during transition care; GHD: growth-hormone deficiency; GHT: growth hormone treatment.

Figure 1 shows the patients’ flowchart. Of 286 patients assessed for eligibility, 124 were excluded, leaving 162 patients for study inclusion. Table 1 reports their main features. The two evaluations were done about 6 years apart in most patients (6.08 years (3.33–10.25)). Of the 162 patients, 105 (64.8%) received GHT for more than 6 months (median, 3.6 years (2.0–6.3)). Of the remaining 57 (35.2%) patients, 51 received no GHT after the adult-care transfer and 6 received GHT for no longer than 6 months. The main reason for discontinuation of GHT was the lack of motivation for the daily injection treatment.

**Table 1:** Characteristics of the population.

	No GHT group	GHT group	*P*
Age at EVAL1*	20.3 (19.1–21.9)	19.3 (18.3–20.8)	
Age at EVAL 2*	26.8 (23.3–33.3)	25.8 (23.3–30.6)	
Number of years of follow up*	5.9 (3.1–10.8)	6.2 (3.4–10.2)	
Duration of GHT (years)*	/	3.6 (2–6.3)	
Female^†^/male^†^	27 (47.4)/30 (52.6)	63 (60)/42 (40)	0.168
Acquired GHD^†^	37 (64.9)	65 (61.9)	0.85
Congenital GHD^†^	18 (31.6)	36 (34.3)	
Idiopathic GHD^†^	2 (3.5)	4 (3.8)	

* Median (1st quartile–3rd quartile).

^†^ Number (percentage).

EVAL1, first evaluation during transition care; EVAL2, second evaluation at least 6 months after first evaluation; GHD, growth hormone deficiency; GHT, growth hormone treatment.

### Bone mineral density

#### Lumbar spine

Of the 105 patients in the GHT group, 72 (69%) and 54 (51%) had available lumbar spine BMD data from EVAL1 and EVAL2, respectively, and 80 (76%) and 59 (56%) had lumbar spine Z-score from the first and second evaluations. Corresponding values in the no-GHT group (*n* = 57) were 32 (56%) and 30 (53%) for LS BMD, and 46 (81%) and 33 (58%) for LS Z-Score.

**Figure 2:**
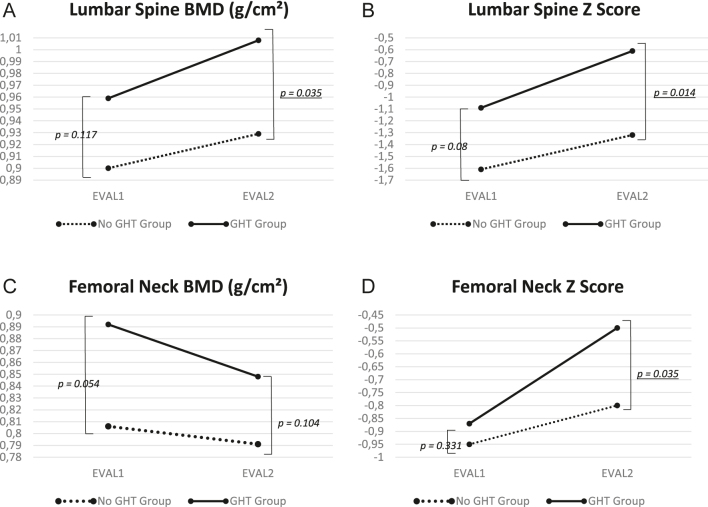
Changes in BMD and Z-score at the lumbar spine and femoral neck between the two evaluations, in the groups with and without growth hormone therapy. EVAL1: first evaluation during transition care; EVAL2: second evaluation at least 6 months after first evaluation; GHT: growth hormone treatment. Statistical analyses within the group between EVAL1 and EVAL2: *lumbar spine BMD in GHT group: *P* = 0.061; lumbar spine BMD in no GHT group: *P* = 0.568. *lumbar spine Z-score in GHT group: *P* = 0.037; lumbar spine BMD in no GHT group: *P* = 0.659. *Femoral neck BMD in GHT group: *P* = 0.219; femoral neck BMD in no GHT group: *P* = 0.439. *Femoral neck Z-score in GHT group: *P* = 0.202; femoral neck BMD in no GHT group: *P* = 0.754.

At the lumbar spine, the no-GHT group had a median BMD of 0.90 (0.83–0.99) g/cm^2^ at the first evaluation and 0.93 (0.85–1.02) g/cm^2^ at the second evaluation (*P* = 0.568; Fig. 2A). The corresponding Z-score values were −1.61 (−2.21 to −0.67) and −1.32 (−2.20 to −0.60), respectively (*P* = 0.659; Fig. 2B). In the GHT group, the median BMD was 0.96 g/cm^2^ (0.86–1.04) and 1.01 g/cm^2^ (0.88–1.14) at the first and second evaluations, respectively (*P* = 0.061; Fig. 2A). The corresponding median Z-scores were −1.09 (−1.90 to −0.02) and −0.61 (−1.60–0.30), respectively (*P* = 0.037; Fig. 2B). Lumbar-spine BMD and lumbar spine Z-score were significantly higher at the second evaluation in the GHT group than in the no-GHT group (*P* = 0.035 and *P* = 0.014, respectively).

For each bone parameter at the lumbar spine, we compared the changes between the two evaluations in the patients with data for both. In the no-GHT group, 16 patients had lumbar-spine BMD data and 25 had lumbar-spine Z-score data at both time points. Corresponding numbers in the GHT group were 37 and 42 patients. The median BMD change from the first to the second evaluation was −0.030 (−0.081–0.0125) g/cm^2^ in the no-GHT group and −0.059 (−0.143 to −0.002) g/cm^2^ in the GHT group (*P* = 0.17). The median difference for the Z-score was 0.07 (−0.42–0.2) in the no-GHT group and −0.30 (−0.93–0) in the GHT group (*P* = 0.047).

#### Femoral neck

Of the 105 patients in the GHT group, 72 (69%) and 49 (47%) had available femoral neck BMD data from EVAL1 and EVAL2, respectively, and 78 (74%) and 52 (49%) had femoral neck Z-score from the first and second evaluation. Corresponding values in the no-GHT group (*n* = 57) were 31 (54%) and 28 (49%) for FN BMD, and 46 (81%) and 30 (53%) for FN Z-score.

In the no-GHT group, the median femoral-neck BMD was 0.81 (0.74–0.90) g/cm^2^ and 0.79 (0.71–0.85) g/cm^2^ at the first and second evaluations, respectively (*P* = 0.439; Fig. 2C). The median Z-score was −0.95 (−1.65 to −0.1) at the first and −0.80 (−1.30 to −0.5) at the second evaluation (*P* = 0.754; Fig. 2D). In the GHT group, the median BMD was 0.89 (0.76–0.98) g/cm^2^ at the first and 0.85 (0.74–0.91) g/cm^2^ at the second evaluation (*P* = 0.219; Fig. 2C). The median Z-scores were −0.87 (−1.50 to 0.18) and −0.50 (−1.33–0.43) at these two time points, respectively (*P* = 0.202; Fig. 2D).

In the no-GHT group, for the femoral neck, 14 patients had BMD data and 23 patients had Z-score data at both time points. Corresponding numbers in the GHT group were 34 and 35 patients. The median BMD change from the first to the second evaluation was 0.055 (−0.022–0.093) g/cm^2^ in the no-GHT group and 0.034 (−0.015–0.065) g/cm^2^ in the GHT group (*P* = 0.76). The corresponding changes in the Z-score were 0.10 (−0.20–0.40) in the no-GHT group and −0.18 (−0.6–0.31) in the GHT group (*P* = 0.16).

#### BMI-adjusted analyses

We performed statistical analyses adjusted for BMI. The results at all sites, comparing the two groups at EVAL1, were not significant (LS BMD: *P* = 0.56; LS Z-score: *P* = 0.54; FN BMD: *P* = 0.38; FN Z-score: *P* = 0.57). These findings are consistent with the unadjusted analyses.

When analyzing only the patients for whom data were available at both time points and comparing the changes between the two groups, the results remained non-significant, with or without adjustment for BMI (data not shown).

### IGF1 and body mass index

At the first evaluation, the median IGF1 level was 147 (66–234.5) ng/mL in the no-GHT group and 182 (99.5–304.5) ng/mL in the GHT group. The corresponding values at the second evaluation were 137 (70.5–219.5) ng/mL and 137 (82–203) ng/mL, respectively.

The median BMI was significantly higher in the GHT than in the no-GHT group at both time points. The value at the first evaluation was 22.3 (20.5–25.5) in the no-GHT group and 25 (22.0–31.0) in the GHT group (*P* = 0.003). At the second evaluation, the corresponding values were 24.1 (21.2–29.1) kg/m^2^ and 27.2 (22.6–32.6) kg/m^2^ (*P* = 0.018).

### Safety

The usual side effects associated with GH treatment were found, with a low prevalence. In the GHT group, GH was discontinued because of the apparition of seizure in one case, worsening of seizure in one case, and headaches in one case. One suspicious cutaneous lesion appeared in one case, resulting in stopping the GHT. No other side effect was reported.

## Discussion

In our retrospective single-center observational cohort study, lumbar spine Z-score improved significantly in the GHT group, by 0.48 SD, whereas the increase in the no-GHT group was only 0.29 SD. Whereas the median lumbar spine BMD and Z-score were no different between the two groups at EVAL1, there was a significant difference at EVAL2, indicating a benefit of continued GHT. The same results were found with femoral neck Z-score but not with BMD. Median BMI was significantly higher in the GHT group, whereas triglyceride and cholesterol levels were not different between the two groups.

GH makes a major contribution to bone maturation and growth via both direct actions and increased IGF1 and IGF2 production (19, 20, 21). The 40–50% increase in bone mass seen during puberty is ascribable to the effects not only of sex hormones but also of GH and IGF1, whose levels are high during this period (22).

Continuing GHT until peak bone mass is achieved has been recommended for patients with CoGHD (5). However, determining when bone mass reaches its peak may be challenging. In a longitudinal study, bone mass growth in females dropped sharply after 15 years of age, i.e., 2–4 years after menarche, whereas bone growth continued in males throughout adolescence (23). Another longitudinal study obtained similar results and also showed very considerable interindividual variability (24). More recently, it has been suggested that peak bone mass may be achieved only between 20 and 25 years of age (5). This possibility is supported by a study in which the age at peak bone mass attainment was about 22 years in females and 23–26 years in males (25).

Our study supports previous findings in smaller populations. In a multicenter randomized controlled trial, 15 patients received GHT in a dosage of 25 μg/kg/d, 15 GHT in a dosage of 12.5 μg/kg/d, and 15 a placebo (26). The patients were adults who had received GHT for CoGHD but had stopped the treatment for at least 1 year (mean, 5.6 years) and had persistent GHD. The lumbar-spine BMD increase was significantly greater with GHT, and a marked dose–response was evidenced. Another randomized trial, in 160 patients, demonstrated significantly greater BMD gains at both the lumbar spine and the hip (13). A longitudinal study compared 16 patients with CoGHT whose treatment was stopped when final height was achieved to 157 controls (27). Peak bone mass was significantly lower in the patients. Moreover, the patients had a rapid decrease in lumbar-spine BMD over the 2 years following attainment of peak bone mass. Thus, the available data support continuation of GHT into adulthood in patients with CoGHD and persistent inadequate GH secretion.

In one study, 113 adolescents who were near final height were taken off GHT then retested 6 months later (28). No adverse bone changes occurred over the 6 months off treatment, perhaps because only 19 patients were found upon retesting to have persistent severe GHD. A small randomized controlled trial studied patients with CoGHD who were taken off GHT when near final height and tested for persistent GHD (29). Of the 40 patients with persistent GHD, 15 were given a placebo and 25 about 20 μg/kg/day of GH. Two years later, the two groups showed no significant differences in bone parameters, body composition, or quality of life. This finding might suggest poor adherence to GHT, but the IGF1 levels were considerably higher with GHT than with the placebo. The younger age at GHT discontinuation than in studies showing benefits of continued GHT might explain the results, and further follow-up data from these patients are needed. A major methodological flaw of this study is the absence of a sample size estimation; given the small number of patients, the absence of statistically significant differences between groups may merely reflect insufficient statistical power. Obstacles to comparisons of published studies include variations in the methods and criteria used to diagnose persistent GHD, GHT dosages used, and treatment adherence (2). In addition, patients with CoGHD have smaller bones than do healthy children, and the areal BMD values provided by dual-energy absorptiometry may therefore underestimate bone mineralization (30). However, this would not affect comparisons between patients with CoGHD who continue GHT and those who discontinue treatment. Bone parameters are affected by numerous factors, such as diet, BMI, and physical activity, which were not controlled for in studies of stopping vs continuing GHT at the transition.

One of the strengths of our study is the long-period follow-up, with an average of 7.3 years and a maximum of 23.7 years. Another strength is that we evaluated a certain number of indicators, such as BMD, Z-Score, and metabolic parameters. Furthermore, the real-life design of this study reflects what we can expect of GH treatment benefit for real patients. A major limitation of our study is the retrospective design. Although the total cohort was large, only small subgroups of patients had bone parameter data at both evaluations, maybe because the recommended interval between BMD evaluations is 2–3 years when previous findings were abnormal and 5 years otherwise (14). Second, adherence to GHT was not measured. The similar IGF1 levels at the second evaluation in the two groups suggest poor adherence in the GHT group. Third, the GHT dosage was not analyzed, which can influence IGF1 level. Fourth, the higher BMI in the GHT group may have contributed to the beneficial effects on bone. In fact, the BMI at the first evaluation was statistically different in the two groups, with a higher BMI in the GHT group, but neither the LS BMD nor the FN BMD was significantly higher in the GHT group. The multivariate analyses adjusted for BMI do not show any differences compared with the univariate analyses. When we compared the changes between the two assessments in the two groups, including only patients with data available at both time points, we did not observe any significant differences, even after adjusting for BMI. This suggests that, although BMI plays a role in bone mineralization, bone health is influenced by multiple factors, and GH treatment in itself contributes to this process. Finally, the single-center recruitment may limit the general applicability of our findings.

In conclusion, our findings support beneficial effects on bone of continuing GHT in patients with CoGHD and persistent GHD during the transition and in early adulthood. The modest nature of the effects in our study may be ascribable to insufficient adherence. Randomized trials with longer follow-ups, standardization of GHD diagnostic methods, careful monitoring of adherence, and correction for confounding by other factors that affect bone are needed to determine whether continued GHT at and after the transition is beneficial.

## Declaration of interest

C Bailly and E Le Roux have no declaration of interest. M Polak declares this conflicts of interest: advisory boards: Ipsen, Novo Nordisk, Pfizer; speaker fees: Novo Nordisk, Pfizer, and Ipsen; research support: Ipsen, Novo Nordisk, Pfizer, Sandoz, Merck, and Sanofi, as well as French institutional grants (PHRC and ANR). P Touraine declares this conflict of interest: consultancy fees from Pfizer. Philippe Touraine is a Senior Editor of *Endocrine Connections*. Philippe Touraine was not involved in the review or editorial process for this paper, on which he is listed as an author.

## Funding

This work was supported by a fund from Pfizer Ink.
